# Vascular aging biomarkers: the role of ADMA, SDMA, and inflammation

**DOI:** 10.3389/fcvm.2026.1922813

**Published:** 2026-10-08

**Authors:** Aleksandra Melnitskaia, Liubov Machekhina, Valentina Pykhtina, Alexey Churov, Olga Maltseva, Wu Zhenghi, Olga Tkacheva, Irina Strazhesko

**Affiliations:** 1 Russian Gerontological Research and Clinical Center, Moscow, Russia; 2 Department of Neurology, Shenzhen Institute of Translational Medicine, The First Affiliated Hospital of Shenzhen University, Shenzhen Second People’s Hospital, Shenzhen, China

**Keywords:** asymmetric dimethylarginine (ADMA), early vascular aging, endothelial dysfunction, high-sensitivity C-reactive protein (hsCRP), interleukin-6 (IL-6), subclinical atherosclerosis, symmetric dimethylarginine (SDMA), vascular aging

## Abstract

Cardiovascular risk assessment based on traditional risk factors may not fully capture subclinical vascular injury and vascular aging, supporting interest in biomarkers reflecting underlying vascular mechanisms. Endothelial dysfunction, altered methylarginine metabolism, and chronic low-grade inflammation represent interconnected processes that may contribute to vascular aging. This narrative review summarizes evidence on the associations of asymmetric dimethylarginine, symmetric dimethylarginine, interleukin-6, and high-sensitivity C-reactive protein with subclinical atherosclerosis and vascular aging. PubMed and Google Scholar were searched between January and August 2026, with priority given to studies evaluating associations of these biomarkers with subclinical vascular changes, vascular aging, and cardiovascular outcomes. ADMA shows the most consistent associations with subclinical atherosclerosis, although these vary across arterial territories and vascular measures. Evidence for SDMA in early vascular disease is more limited, and interpretation is complicated by its dependence on renal function, although some associations persist after adjustment for renal function. IL-6 has biological and genetic evidence supporting a role in atherogenesis, but its associations with subclinical atherosclerosis are not consistently independent of traditional cardiovascular risk factors. hsCRP is an established cardiovascular risk marker and has also been associated with progression of carotid stenosis and arterial stiffness. Differences in vascular endpoints, study populations, confounder adjustment, and analytical methods contribute to heterogeneity across studies. Overall, these biomarkers appear to reflect partly distinct but interconnected components of vascular aging. Their potential relevance may lie in their complementarity; however, current evidence does not establish that combined assessment improves evaluation beyond conventional approaches. Prospective studies should determine whether their combined assessment improves identification of accelerated vascular aging beyond conventional risk assessment.

## Introduction

Cardiovascular diseases (CVDs) remain the leading cause of mortality worldwide. According to recent global estimates, CVDs accounted for approximately 19 million deaths in 2022, with projections suggesting an increase to 35.6 million deaths by 2050 (1, 2). Coronary artery disease (CAD) continues to be the principal contributor to cardiovascular mortality, with a substantial proportion of cases occurring in working-age individuals, underscoring the major socioeconomic burden of the disease (3, 4).

Atherosclerotic injury to the arterial wall underlies most CVDs (5). However, the clinical manifestations and progression of atherosclerosis are highly heterogeneous, and some individuals exhibit accelerated vascular deterioration. This observation supports the concept of atherosclerosis as an age-associated condition linked to premature biological aging (6–8).

The concept of vascular aging originated from findings of the Framingham Heart Study and was later expanded by P. Nilsson through the introduction of the term “early vascular aging” (EVA) (9). EVA is defined as a mismatch between chronological age and vascular wall status, whereby arterial stiffness, pulse wave velocity, and related vascular parameters correspond to those typically observed in older individuals (10). Within this framework, three vascular aging phenotypes are currently recognized: early (EVA), normal, and supernormal vascular aging (SUPERNOVA) (11).

Traditional cardiovascular risk factors including hypertension, dyslipidemia, smoking, and diabetes mellitus remain important predictors of cardiovascular events in clinical practice and form the basis of most risk prediction models (12, 13). However, estimated cardiovascular risk does not necessarily reflect the presence of subclinical vascular injury. Indeed, vascular abnormalities have been observed even among individuals classified as having low or intermediate cardiovascular risk (14). Moreover, the performance of conventional risk prediction models may vary across populations, potentially limiting the accuracy of individual risk estimation (15). These limitations support the investigation of additional pathophysiologically relevant biomarkers that may complement conventional risk assessment by identifying early vascular changes and characterizing vascular biological aging.

Within the endothelial-inflammatory continuum, particular attention has been directed toward biomarkers reflecting endothelial function and chronic low-grade inflammation. The methylarginines asymmetric dimethylarginine (ADMA) and symmetric dimethylarginine (SDMA) may impair nitric oxide bioavailability through distinct mechanisms: ADMA acts as a direct endogenous inhibitor of nitric oxide synthase, whereas SDMA may reduce intracellular L-arginine availability by competing for cationic amino acid transport (16, 17). Proinflammatory mediators such as interleukin-6 (IL-6) contribute to endothelial activation and a proinflammatory vascular phenotype, whereas high-sensitivity C-reactive protein (hsCRP) primarily serves as a circulating marker of systemic inflammation (5).

A recent systematic review specifically addressing the role of methylarginines in vascular aging identified only a small number of studies meeting the inclusion criteria, highlighting the limited and heterogeneous nature of direct evidence in this field (18). However, methylarginines are closely linked to the endothelial component of vascular aging, whereas endothelial dysfunction develops in close interplay with chronic low-grade inflammation. Therefore, considering methylarginines together with inflammatory biomarkers may provide a more comprehensive understanding of the mechanisms and early manifestations of vascular aging.

Given the limited direct evidence, coronary artery calcification (CAC), carotid intima-media thickness (cIMT), and carotid plaque are considered in this review as indirect correlates of vascular aging and subclinical atherosclerosis rather than specific measures of vascular aging.

The aim of this narrative review was to summarize current evidence on the associations of ADMA, SDMA, IL-6, and hsCRP with markers of early atherosclerosis and their potential relevance to premature vascular aging. The novelty of this review lies in considering these biomarkers not as isolated indicators, but as complementary markers of interconnected endothelial and inflammatory processes that may contribute to accelerated vascular aging.

## Materials and methods

In this narrative review, the literature search was conducted between January and August 2026 using PubMed and Google Scholar. English-language studies published between 2000 and 2026 were considered. For the clinical evidence synthesis, studies involving adult populations (>18 years) were eligible for inclusion, with a particular focus on subclinical atherosclerosis and vascular aging. Meta-analyses, prospective cohort studies, randomized clinical trials, and other observational studies were primarily considered for the evaluation of clinical evidence. Selected experimental and translational studies were additionally included to provide mechanistic context for the relationships between the investigated biomarkers, endothelial dysfunction, inflammation, and vascular aging.

The PubMed search comprised four sequential searches focused on ADMA, SDMA, hsCRP, and IL-6. For each biomarker, Medical Subject Headings (MeSH) and free-text terms were combined using Boolean operators with terms related to vascular aging and subclinical atherosclerosis (“vascular aging”, “vascular ageing”, “arterial stiffness”, “arteriosclerosis”, “early atherosclerosis”, “atherosclerosis”), cardiovascular disease (“cardiovascular disease”, “CVD”), and endothelial function (“endothelial dysfunction”, “endothelial function”, “vascular endothelium”, “endothelial cells”). Biomarker-specific terms included “asymmetric dimethylarginine” or “ADMA”; “symmetric dimethylarginine” or “SDMA”; “high-sensitivity C-reactive protein”, “hs-CRP” or “CRP”; and “interleukin-6”, “IL-6” or “IL6”. The search was restricted to human studies for the clinical evidence synthesis, and eligibility was subsequently assessed according to the inclusion criteria described above.

Google Scholar was additionally searched using combinations of the same biomarker-specific terms (ADMA, SDMA, hsCRP, and IL-6) with terms related to vascular aging, atherosclerosis, arterial stiffness, and endothelial dysfunction. Reference lists of relevant reviews and selected studies were also screened to identify additional publications of potential relevance.

Studies were selected based on their relevance to the biological mechanisms and clinical associations discussed in this review rather than through a formal systematic review process. Particular attention was given to studies evaluating associations between the biomarkers of interest and measures of subclinical vascular injury, including carotid intima-media thickness, coronary artery calcification, and arterial stiffness, as well as to studies providing mechanistic evidence linking these biomarkers to endothelial dysfunction, inflammation, and vascular aging.

### Biology and pathophysiology of key biomarkers

Asymmetric and symmetric dimethylarginines are generated through methylation of arginine residues by protein arginine methyltransferases (PRMTs), followed by proteolysis. Their regulation differs substantially. Approximately 70%–90% of ADMA is metabolized by dimethylarginine dimethylaminohydrolase (DDAH), yielding L-citrulline and dimethylamine (16). In contrast, SDMA undergoes minimal metabolic degradation and is primarily excreted by the kidneys, resulting in a close relationship between circulating SDMA concentrations and renal function, including glomerular filtration rate (GFR) (19).

ADMA acts as an endogenous competitive inhibitor of endothelial nitric oxide synthase (eNOS), reducing the production of nitric oxide (NO), a key vasoprotective mediator. The balance between L-arginine availability and ADMA is therefore an important determinant of NO synthesis, and a reduced L-arginine/ADMA ratio may contribute to impaired endothelial NO bioavailability (16). Elevated ADMA may promote eNOS uncoupling, resulting in increased superoxide generation and reduced NO production and thereby shifting the NO/ROS balance toward oxidative stress (20). Reduced NO bioavailability, together with increased ROS production, promotes vasoconstriction and endothelial dysfunction. Clinically, elevated ADMA levels have been associated with impaired endothelium-dependent vasodilation and increased cardiovascular risk (21).

Unlike ADMA, SDMA does not directly inhibit eNOS. However, it competes with L-arginine for transport via cationic amino acid transporters, thereby reducing intracellular substrate availability for NO synthesis (16). Elevated SDMA levels are commonly observed in individuals with reduced glomerular filtration rate and chronic kidney disease (22). In addition, experimental studies summarized by Riddell et al. suggest that SDMA promotes the production of reactive oxygen species and proinflammatory cytokines, including IL-6 and TNF-α, thereby potentially linking SDMA to both oxidative stress and vascular inflammation (23).

Oxidative and metabolic stress further amplify endothelial dysfunction through activation of inflammatory pathways. In particular, excessive ROS production and reduced NO bioavailability can interact with activation of the NLRP3 inflammasome, linking oxidative stress to endothelial inflammation and injury (24). Metabolic disturbances such as insulin resistance and lipotoxicity may further impair endothelial NO signaling and promote inflammatory activation. Recent experimental evidence in human coronary artery endothelial cells showed that oxidative and lipotoxic stress impaired endothelial cell viability and angiogenic function, whereas pharmacological NLRP3 inhibition attenuated these effects, supporting a mechanistic role for inflammasome activation in endothelial injury (25).

IL-6 may contribute to endothelial dysfunction by impairing eNOS-dependent NO signaling. Experimental studies in human endothelial cells have shown that IL-6 reduces eNOS phosphorylation at Ser1177 and decreases NO production through several mechanisms, including interference with insulin-mediated endothelial signaling (26, 27). In parallel, IL-6 trans-signaling activates proinflammatory pathways in vascular endothelial cells, including JAK/STAT3 and PI3 K/AKT signaling, promoting MCP-1 production and increased expression of the adhesion molecules VCAM-1 and ICAM-1 (28). Together, these mechanisms may promote endothelial activation, leukocyte recruitment, and a proinflammatory vascular phenotype.

IL-6 also stimulates hepatic production of high-sensitivity C-reactive protein, forming the IL-6–hsCRP inflammator*y* axis. Elevated hsCRP therefore provides an integrated marker of downstream systemic inflammatory activity, although its direct causal contribution to endothelial dysfunction and vascular injury remains uncertain.

Taken together, ADMA, SDMA, IL-6, and hsCRP participate in an interconnected network linking impaired NO signaling, oxidative stress, and inflammation. Inflammation and oxidative stress suppress DDAH activity, thereby promoting ADMA accumulation, while excess ADMA further reduces NO bioavailability and favors ROS generation. SDMA may additionally limit intracellular L-arginine availability and promote inflammatory signaling. In parallel, metabolic stress, NLRP3 inflammasome activation, and IL-6/hsCRP-mediated inflammation can sustain endothelial activation and injury. These mutually reinforcing pathways shift the endothelial NO/ROS balance toward oxidative stress and may impair endothelial homeostasis, thereby contributing to vascular aging and atherogenesis (Figure 1).

**Figure 1:**
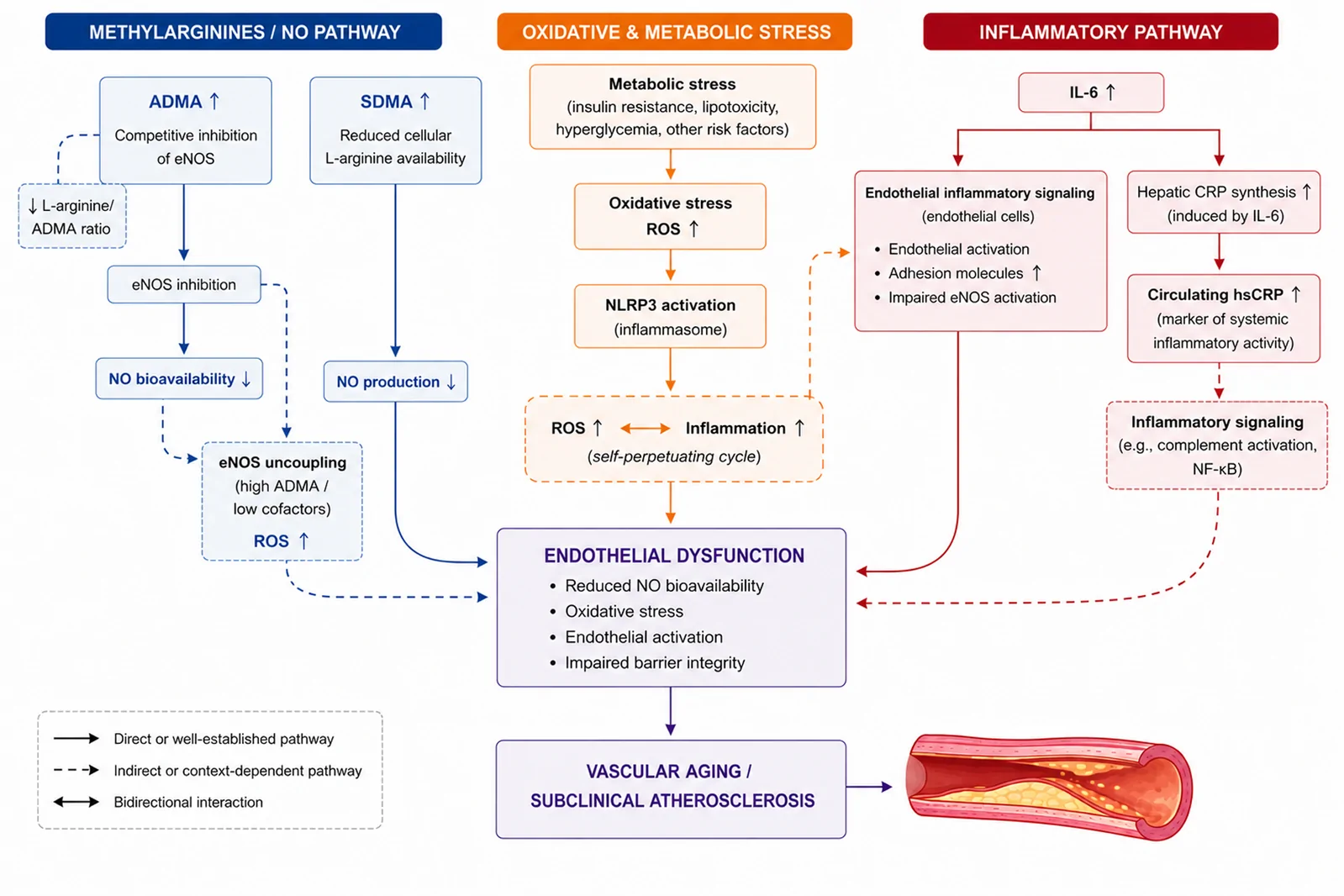
Proposed endothelial-inflammatory framework linking ADMA, SDMA#, IL-6, and hsCRP to vascular aging and subclinical atherosclerosis. ADMA and SDMA impair NO-dependent endothelial homeostasis through inhibition of eNOS and reduced cellular L-arginine availability, respectively. Oxidative and metabolic stress promotes ROS generation and NLRP3 inflammasome activation, reinforcing the interaction between oxidative stress and inflammation. IL-6 contributes to endothelial inflammatory activation and stimulates hepatic CRP synthesis, whereas circulating hsCRP primarily reflects systemic inflammatory activity. Together, impaired NO signaling, oxidative stress, and inflammation contribute to endothelial dysfunction and may promote vascular aging and subclinical atherosclerosis. Solid arrows indicate direct or well-established pathways, dashed arrows indicate indirect or context-dependent pathways, and bidirectional arrows indicate mutually reinforcing interactions. ADMA, asymmetric dimethylarginine; SDMA, symmetric dimethylarginine; eNOS, endothelial nitric oxide synthase; NO, nitric oxide; ROS, reactive oxygen species; IL-6, interleukin-6; hsCRP, high-sensitivity C-reactive protein; CRP, C-reactive protein; NLRP3, NOD-like receptor family pyrin domain-containing 3.

### Clinical manifestations of the endothelial-inflammatory continuum: associations between biomarkers and subclinical atherosclerosis

#### ADMA as an integrated marker of endothelial dysfunction and early atherogenesis

Among the biomarkers discussed, ADMA has demonstrated the most consistent association with subclinical atherosclerosis. Elevated ADMA levels were independently and significantly associated with carotid intima-media thickness (IMT), even after adjustment for age, sex, and systolic blood pressure. Tertile analysis further demonstrated a linear relationship between IMT and circulating ADMA concentrations (*p* < 0.001) (29). These findings are supported by a meta-analysis including more than 6000 participants, in which ADMA remained independently associated with IMT after adjustment for potential confounders (PR = 0.21; 95% CI, 0.14–0.29; *p* < 0.001). Notably, this association was particularly pronounced in patients with chronic kidney disease (30).

Data from the Framingham Heart Study further suggest segment-specific vascular associations. After multivariable adjustment, ADMA was not associated with common carotid artery IMT (*p* = 0.991), but remained significantly associated with IMT of the internal carotid artery and carotid bifurcation (*β*=0.0246; *p* = 0.002), vascular segments known to be more susceptible to atherosclerotic plaque formation (31). These segment-specific associations suggest that ADMA may be more closely related to focal atherosclerotic changes than to diffuse vascular remodeling.

The association between ADMA and coronary artery calcification further supports its role in early atherosclerosis. In the offspring cohort of the Framingham Heart Study, higher ADMA levels were associated with increased coronary artery calcification (*β*=0.25; 95% CI, 0.11–0.39, *p* = 0.001) (32).

Clinical studies in populations at increased risk of accelerated vascular aging further reinforce these observations. In patients with chronic kidney disease (CKD), ADMA has been associated with markers of subclinical vascular disease. In a cohort of 93 patients with chronic non-diabetic renal insufficiency without clinically overt atherosclerosis, plasma ADMA concentrations demonstrated a strong association with carotid IMT in both univariate (*β*=0.459; *p* < 0.0001) and multivariable analyses (*β*=0.444; *p* < 0.0001) (33). These findings support an association between elevated ADMA and subclinical vascular changes before clinically overt atherosclerotic disease.

In another CKD cohort, ADMA levels were associated with progression of the coronary artery calcium score (CACS) in univariate analysis (r = 0.389; *p* = 0.005). In the multivariable model incorporating potential confounders, only ADMA remained independently associated with CACS progression (*p* = 0.048), whereas associations with diabetes, estimated glomerular filtration rate, serum calcium, and phosphate levels did not reach statistical significance (34).

Among individuals with stable chronic obstructive pulmonary disease (COPD) and no overt cardiovascular disease, ADMA levels positively correlated with brachial intima-media thickness (r = 0.331; *p* = 0.013). In multivariable analysis, ADMA remained an independent predictor of brachial intima-media thickness (standardized *β*=0.311; *p* = 0.011), supporting its association with subclinical vascular changes even outside classical cardiovascular settings (35). In contrast, in antiretroviral therapy-treated individuals with HIV infection, ADMA was not associated with coronary artery calcium score, myocardial perfusion defects, or carotid IMT (36).

Interventional studies provide additional evidence that changes in ADMA may accompany favorable changes in vascular phenotype. In a randomized study of individuals with stage 1 hypertension and no prior cardiovascular disease, a 6-month lifestyle intervention resulted in a significant reduction in ADMA levels (*p* ≤ 0.001), accompanied by improvements in arterial stiffness and carotid IMT. Changes in the dietary inflammatory index were associated with changes in ADMA levels (*β*=0.089; 95% CI, 0.069–0.109; *p* ≤ 0.001), further supporting a link between the inflammatory potential of diet and endothelial dysfunction (37).

#### SDMA as a marker of vascular injury

In populations without clinically overt cardiovascular disease, data regarding SDMA remain limited, restricting assessment of its role during the early stages of vascular injury. In the Dallas Heart Study, a multiethnic population-based cohort of adults aged 30–65 years, both SDMA and ADMA were associated with coronary artery calcium >10 Agatston units in unadjusted analyses. However, after adjustment for age, sex, race, traditional cardiovascular risk factors, and renal function, only SDMA remained independently associated with coronary artery calcium >10, whereas abdominal aortic wall thickness remained associated with both SDMA and ADMA (38).

Population-based data from the Study of Health in Pomerania (SHIP) provide additional evidence for an association between SDMA and subclinical vascular changes. In 1,999 participants, higher SDMA concentrations were associated with increased carotid intima-media thickness after multivariable adjustment, including adjustment for estimated glomerular filtration rate (eGFR), whereas no independent association was observed with carotid plaque (39).

Given the limited evidence in preclinical populations, studies involving high-risk cohorts may provide indirect insight into the vascular and prognostic relevance of SDMA. In patients with symptomatic peripheral artery disease, elevated levels of both ADMA and SDMA were independently associated with increased cardiovascular mortality, with threshold values of >0.745 μmol/L and >0.825 μmol/L, respectively (*p* < 0.001 and *p* = 0.030). These associations were observed in the context of advanced vascular injury, including impaired endothelium-dependent vasodilation and increased brachial intima-media thickness, supporting a link between methylated arginines and adverse cardiovascular outcomes (40).

Collectively, these findings suggest that the prognostic significance of SDMA may be most pronounced in the presence of established or clinically manifest atherosclerosis, whereas its role during preclinical stages remains less clearly defined. In this context, SDMA may primarily reflect systemic vascular injury and remodeling, whereas ADMA appears to be more closely related to earlier stages of endothelial dysfunction.

The strength of these associations may also depend on the clinical context. In patients with rheumatoid arthritis, no significant overall associations between dimethylarginines and functional or structural vascular measures were observed. However, significant interactions with inflammatory status indicated stronger associations between selected dimethylarginine measures and vascular parameters in patients with higher levels of inflammatory markers (41). These findings suggest that inflammatory status may modify the vascular associations of dimethylarginines and underscore the need for cautious interpretation of SDMA across different clinical populations.

#### Inflammatory markers (IL-6 and high-sensitivity CRP) and subclinical atherosclerosis

Chronic low-grade inflammation is an important component of vascular aging, with IL-6 representing one of its key circulating markers. Elevated IL-6 levels have been reported across a range of age-related diseases (42). Genetic evidence further supports a potential causal role of IL-6 signaling in atherogenesis: IL6R variants associated with reduced IL-6 signaling have been linked to a lower risk of coronary heart disease (43). Notably, associations between circulating IL-6 and cardiovascular and vascular imaging outcomes have been reported across diverse populations, including after adjustment for hsCRP, supporting the concept that IL-6 may reflect a more upstream component of the inflammatory cascade (44).

Clinical evidence further supports the relevance of IL-6. In patients at intermediate cardiovascular risk referred for coronary angiography, serum IL-6 levels above 1 pg/mL were identified as a significant predictor of coronary artery disease [AUC 0.74, 95% CI (0.57–0.84)], demonstrating superior predictive performance compared with hsCRP (45).

Data from the Multi-Ethnic Study of Atherosclerosis (MESA) further illustrate the association between IL-6 and coronary artery calcification. In a cross-sectional analysis of 6,783 participants without clinically apparent cardiovascular disease, participants in the highest versus lowest quartile of IL-6 had a higher prevalence of detectable CAC, and this association remained significant after adjustment for traditional cardiovascular risk factors (RR = 1.12; 95% CI, 1.06-1.20; *p* < 0.01) (46).

Longitudinal MESA data subsequently showed that higher IL-6 levels were associated with incident CAC among participants without CAC at baseline (RR = 1.09; *p* = 0.010) and with CAC progression among those with prevalent CAC (*β*=2.9; *p* < 0.05) in minimally adjusted models. However, these associations were attenuated and no longer statistically significant after further adjustment for traditional cardiovascular risk factors and statin use (47).

Whereas IL-6 may represent a more upstream mediator of vascular inflammation, hsCRP is a downstream and more widely studied circulating marker of systemic inflammatory activity that has been extensively evaluated in large cardiovascular cohorts. In the large prospective Women's Health Study of 27,939 initially healthy women, higher baseline hsCRP levels predicted cardiovascular events over 30 years, with an adjusted HR of 1.70 (95% CI, 1.52–1.90) for the highest versus lowest quintile (48). Although CRP is not a specific marker of atherosclerosis, elevated hsCRP levels have also been associated with subclinical vascular injury and its progression, as discussed below.

A prospective study of 1,268 patients initially asymptomatic with respect to carotid artery disease demonstrated that higher hsCRP levels at baseline and follow-up, as well as increases in hsCRP over time, were significantly associated with progression of carotid atherosclerosis (49). In the prospective Kailuan cohort study of 25,336 participants, higher baseline hsCRP levels were associated with greater increases in arterial stiffness, assessed by brachial-ankle pulse wave velocity (baPWV), over a mean follow-up of 4.62 years. After multivariable adjustment, compared with participants with hsCRP <1 mg/L, increases in baPWV were greater by 14.44 cm/s in those with hsCRP levels of 1–3 mg/L and by 40.67 cm/s in those with hsCRP >3 mg/L (both *p* < 0.001), suggesting a graded association between hsCRP levels and progression of arterial stiffness (50). More recently, the Rotterdam Study found that hsCRP mediated a small proportion of the association between age and both cIMT (2.66%) and PWV (2.56%). Mendelian randomization supported a potential causal relationship between hsCRP and PWV, but not cIMT, suggesting that inflammation may contribute differently to functional arterial stiffening and structural vascular remodeling (51). Key studies examining the associations of ADMA, SDMA, IL-6, and hsCRP with subclinical vascular phenotypes are summarized in Table 1.

**Table 1:** Clinical evidence linking ADMA, SDMA, IL-6, and hsCRP to subclinical vascular injury and vascular aging.

Biomarker	Study, year	Population/study design	Vascular endpoint	Main findings	Adjusted confounders
ADMA	**Furuki et al., 2007** (29)	*n* = 712; community-based cross-sectional study; participants >40 years without overt cerebro-cardiovascular disease	Common carotid IMT	ADMA was independently associated with carotid IMT (*p* < 0.01); IMT increased linearly across ADMA tertiles after adjustment	Age, sex, and other cardiovascular confounders; in stepwise multiple regression, ADMA, age, and SBP were independent determinants of IMT.
ADMA	**Maas et al., 2009** (31)	Framingham Offspring Study; *n* = 2,958; cross-sectional	CCA-IMT; ICA/bulb-IMT	ADMA was not associated with CCA-IMT after adjustment (*p* = 0.991), but remained associated with ICA/bulb-IMT (*β* per SD = 0.0246; *p* = 0.002)	Age, sex, SBP, antihypertensive treatment, smoking, diabetes, BMI, total/HDL cholesterol ratio, log-CRP, serum creatinine
ADMA	**Bai et al., 2013** (30)	Systematic review/meta-analysis; 22 studies; *n* = 6,168	Carotid IMT	ADMA was positively associated with carotid IMT (pooled R = 0.29; pooled adjusted partial R = 0.21); association was stronger in CKD populations	Study-specific adjustment; pooled partial correlation coefficients were based on adjusted estimates
ADMA	**Castro-Diehl et al., 2021** (32)	Framingham Offspring Study; *n* = 890 for CAC; *n* = 1,697 for IMT; cross-sectional vascular analyses	CAC; carotid IMT	Higher ADMA was independently associated with CAC (β per SD = 0.25; 95% CI 0.11–0.39), but not with carotid IMT (β per SD = 0.02; 95% CI −0.02-0.05).	Age, sex, BMI, SBP, antihypertensive medication, diabetes, current smoking, total/HDL cholesterol ratio; eGFR was not included in the subclinical atherosclerosis models
SDMA/ADMA	**Gore et al., 2013** (38)	Dallas Heart Study; *n* = 3,523 with ADMA/SDMA measurements; multiethnic population-based cohort	CAC >10; abdominal aortic wall thickness	After adjustment for conventional risk factors and GFR, SDMA remained associated with CAC >10, whereas ADMA did not. In the full-text vascular analyses, aortic wall thickness was associated with both SDMA and ADMA.	Age, sex, race/ethnicity; diabetes, BMI, hypertension, hypercholesterolemia, current smoking, previous MI or chronic heart failure; GFR
SDMA	**Bahls et al., 2015** (39)	SHIP; *n* = 1,999; population-based cross-sectional study	cIMT; carotid plaque	High SDMA was associated with increased cIMT (>75th age- and sex-specific percentile; OR 1.39; 95% CI 1.08–1.79); no independent association was observed with carotid plaque; ADMA was not associated with either vascular measure.	Sex, age, smoking status, waist-to-hip ratio, eGFR
IL-6	**Wainstein et al., 2017** (45)	*n* = 48; intermediate-risk patients with chronic chest pain referred for elective coronary angiography; cross-sectional	Angiographic CAD (≥30% stenosis)	IL-6 > 1 pg/mL was associated with CAD; full-text results report AUC 0.74 (95% CI 0.57–0.84), versus 0.60 for hsCRP and 0.54 for ASCVD score	No multivariable adjustment for confounders. Major exclusions included diabetes and eGFR <45 mL/min/1.73 m^2^
IL-6	**Jenny et al., 2010** (46)	MESA; *n* = 6,783; multiethnic cohort free of clinical CVD; cross-sectional	CAC presence	After full adjustment, IL-6 remained associated with CAC presence (highest vs lowest quartile RR 1.12; 95% CI 1.06–1.20); CRP was no longer significant (RR 1.05; 95% CI 0.99–1.12)	Age, sex, ethnicity, smoking, diabetes, blood pressure, obesity, dyslipidemia
IL-6	**Zeb et al., 2021** (47)	MESA; longitudinal cohort; 3,115 participants with CAC=0 and 2,896 with CAC>0 at baseline; mean follow-up 6.5 years	Incident CAC; CAC progression	IL-6 was associated with incident CAC (RR = 1.09; *p* = 0.010) and CAC progression (β=2.9; *p* < 0.05) in minimally adjusted models; associations were no longer significant after full adjustment.	Minimally adjusted: follow-up time, age, sex, race/ethnicity (incident CAC); scanner type, age, sex, race/ethnicity (CAC progression). Fully adjusted: additional traditional cardiovascular risk factors and statin use.
hsCRP	**Schillinger et al., 2005** (49)	ICARAS; *n* = 1,268; prospective study; median follow-up 7.5 months	**Progression of carotid stenosis**, assessed by serial duplex ultrasound	Baseline and follow-up hsCRP, and increases in hsCRP over follow-up, were associated with progression; highest vs. lowest baseline hsCRP quintile adjusted OR 3.65 (95% CI 1.65–8.08)	Age, sex, BMI, HbA1c, current smoking, hypertension, LDL cholesterol, family history of CVD, PAD, previous MI, previous stroke, serum creatinine, statin therapy, baseline stenosis; clustering by side of lesion
hsCRP	**Li et al., 2025** (50)	Kailuan prospective cohort; *n* = 25,336 with ≥2 baPWV measurements; mean interval 4.62 ± 2.86 years	Longitudinal change in baPWV	Compared with hsCRP <1 mg/L, adjusted differences in longitudinal baPWV change were 14.44 cm/s (95% CI 6.54–22.34) for hsCRP 1–3 mg/L and 40.67 cm/s (95% CI 5.28–51.02) for hsCRP >3 mg/L	Age, sex, follow-up time, MAP, LDL-C, HDL-C, total cholesterol, triglycerides, fasting glucose, resting heart rate, BMI, smoking, alcohol intake, physical activity
hsCRP	**Mohammadi Jouabadi et al., 2026** (51)	Rotterdam Study; *n* = 7,591 for **longitudinal cIMT**; *n* = 6,488 for **cross-sectional PWV**; mediation analysis + two-sample MR	cIMT; PWV	hsCRP mediated 2.66% of the age–cIMT association and 2.56% of the age–PWV association. MR provided evidence supporting a potential causal association with PWV, but not cIMT	Sex, BMI, prevalent CVD, hypertension, blood pressure-lowering, glucose-lowering, cardiac and lipid-lowering medications; vascular outcome models additionally adjusted for sub-cohort, follow-up time (cIMT), SBP, DBP, and pulse rate.

## Discussion

The evidence reviewed here supports the concept that endothelial dysfunction and chronic low-grade inflammation represent interconnected components of vascular aging. Within this endothelial-inflammatory continuum, ADMA is closely linked to impaired nitric oxide signaling and endothelial dysfunction, whereas SDMA may reflect altered L-arginine availability, renal function, and vascular injury. IL-6 and hsCRP characterize different aspects of systemic inflammatory activity. These biomarkers therefore represent partly distinct but biologically interconnected pathways and should not be interpreted as interchangeable markers of a single pathological process.

Among the biomarkers considered, ADMA shows the most consistent associations with subclinical atherosclerosis across both population-based and clinical studies. However, the vascular changes associated with ADMA are not uniform. In the Framingham Offspring Study, ADMA was associated with IMT of the internal carotid artery and carotid bifurcation, but not with common carotid IMT, while a subsequent Framingham analysis found an association with coronary artery calcification but not carotid IMT. Together with meta-analytic evidence linking ADMA to carotid IMT, these findings suggest that its associations may vary according to arterial territory and the vascular measure assessed and may be more pronounced for some focal atherosclerotic changes. Nevertheless, most observations are cross-sectional and establish associations rather than temporal or causal relationships. Lifestyle intervention data showing parallel reductions in ADMA and improvements in vascular measures support its biological relevance but do not establish that lowering ADMA mediates vascular improvement.

The interpretation of SDMA is more complex. Although SDMA has been associated with coronary calcification, aortic wall thickness, carotid IMT, and cardiovascular mortality, evidence for early vascular disease is more limited than for ADMA. SDMA is also strongly influenced by renal clearance, complicating interpretation of its vascular associations. Nevertheless, associations of SDMA with coronary calcification in the Dallas Heart Study and with cIMT in SHIP persisted after adjustment for GFR or eGFR, suggesting that these vascular associations cannot be explained solely by impaired renal function. Its strong dependence on renal function, however, complicates assessment of SDMA as an independent marker of vascular injury and aging.

Inflammation provides a potential biological link between methylarginine pathways and vascular injury. Experimental evidence indicates close interactions among impaired NO signaling, oxidative stress, and inflammatory activation, while clinical observations suggest that inflammatory status may influence the associations between dimethylarginines and measures of vascular structure and function. In rheumatoid arthritis, these associations became more apparent in the presence of higher inflammatory activity. These findings are consistent with the concept that endothelial dysfunction and inflammation represent interacting components of vascular injury, but clinical evidence remains insufficient to establish inflammation as a modifier of dimethylarginine-vascular associations. This hypothesis therefore requires prospective validation.

The evidence for IL-6 highlights the distinction between biological relevance and independent associations with vascular outcomes. Genetic studies support a potentially causal role of IL-6 signaling in atherogenesis, while cross-sectional MESA data demonstrated an association between IL-6 and prevalent coronary calcification after adjustment for conventional cardiovascular risk factors. However, associations with incident CAC and CAC progression in longitudinal MESA analyses were no longer significant after further adjustment for cardiovascular risk factors and statin use. Thus, although IL-6 may occupy a relatively upstream position in the inflammatory cascade and contribute to atherogenesis, its associations with subclinical atherosclerosis are not consistently independent of traditional cardiovascular risk factors.

A similar distinction applies to hsCRP. Its association with long-term cardiovascular risk is well established, while the studies reviewed here also link higher hsCRP to progression of carotid stenosis and arterial stiffness. The Kailuan cohort demonstrated a graded relationship between baseline hsCRP and subsequent increases in baPWV, whereas in the Rotterdam Study hsCRP mediated only a small proportion of the association between age and both arterial stiffness and cIMT. Mendelian randomization supported a potential causal relationship between hsCRP and PWV but not cIMT, suggesting that inflammatory pathways may contribute differently to functional arterial stiffening and structural vascular remodeling.

This endpoint-specificity may explain part of the apparent inconsistency across studies. Carotid IMT, focal carotid lesions, CAC, aortic wall thickness, arterial stiffness, and endothelium-dependent vasodilation reflect different aspects of vascular pathology and should therefore be interpreted as indirect correlates rather than specific measures of vascular aging. Even within carotid ultrasonography, common carotid IMT and IMT at the bifurcation or internal carotid artery may reflect partly different vascular processes. Thus, the absence of an association with one vascular measure does not necessarily argue against a biomarker's relevance to vascular aging. Conversely, associations with heterogeneous vascular endpoints do not by themselves establish that a biomarker reflects a common underlying process of vascular aging. Future studies should therefore distinguish more clearly between structural and functional vascular endpoints.

Several methodological issues further limit comparisons between studies. The available evidence includes cross-sectional and prospective population studies, selected clinical populations, and relatively small mechanistic or interventional studies. Biomarker–vascular associations may be influenced by age, renal function, cardiovascular risk factors, medication use, systemic inflammation, and comorbidities, while adjustment for these factors varies substantially across studies. Renal function is particularly important because of its strong relationship with SDMA and its potential to confound methylarginine–vascular associations.

A specific limitation relevant to ADMA and SDMA concerns analytical standardization. These biomarkers can be quantified using HPLC, mass spectrometry-based methods, and immunoassays, and methodological differences may contribute to variability in reported concentrations. Given their relatively narrow circulating concentration ranges, accurate and reproducible measurement is important for comparisons across studies. The lack of standardized measurement methods, together with substantial variability in reported reference ranges for ADMA, limits comparability and remains a barrier to clinical implementation (16, 52).

Another limitation is the distinction between statistical association and incremental clinical utility. Most studies assessed associations with vascular measures after adjustment for selected covariates, but such analyses do not demonstrate that measuring ADMA, SDMA, IL-6, or hsCRP improves assessment of vascular aging or cardiovascular risk beyond established clinical models. Evidence regarding discrimination, calibration, or improvement in risk classification is limited, particularly for ADMA and SDMA. Thus, the available evidence supports the biological relevance and vascular associations of these biomarkers but does not establish their incremental utility for vascular aging assessment. This is particularly relevant in individuals at low or intermediate cardiovascular risk, in whom additional biomarkers would need to improve detection of subclinical vascular disease or prediction of future events beyond conventional assessment.

Taken together, the available evidence suggests that no single biomarker considered here is likely to fully characterize vascular aging, as endothelial NO dysfunction, renal–methylarginine metabolism, upstream inflammatory signaling, and downstream systemic inflammation represent partly distinct but interacting biological components. The potential value of ADMA, SDMA, IL-6, and hsCRP may therefore lie in their complementarity rather than interchangeability. This provides a biological rationale for evaluating these markers in combination to characterize different components of vascular aging. However, whether a combined profile of endothelial and inflammatory biomarkers provides additional clinical value beyond individual biomarkers remains to be tested directly.

Future studies should move beyond cross-sectional associations between biomarkers and vascular measures. Large prospective cohorts with standardized biomarker measurements, repeated assessment of vascular structure and function, and adjudicated cardiovascular outcomes are needed to assess whether these biomarkers predict accelerated vascular aging independently of chronological age and established risk factors. Particular attention should be given to consistent assessment and adjustment for renal function, direct comparison of vascular endpoints, and evaluation across populations with and without cardiovascular or renal disease. Combined biomarker models should be evaluated against established cardiovascular risk models using measures of discrimination, calibration, and reclassification. Such studies would clarify whether the endothelial-inflammatory continuum provides not only a biologically coherent framework for understanding vascular aging but also a basis for clinically useful biomarker assessment.

## Conclusion

The available evidence supports the involvement of endothelial dysfunction, altered methylarginine metabolism, and chronic low-grade inflammation in vascular aging. ADMA, SDMA, IL-6, and hsCRP appear to reflect partly distinct components of these interconnected processes and should not be considered interchangeable markers of vascular aging. ADMA shows the most consistent associations with subclinical atherosclerosis, although these associations vary across arterial territories and vascular measures. Interpretation of SDMA requires particular consideration of renal function, while IL-6 and hsCRP appear to reflect different aspects of inflammatory activity.

Taken together, these findings suggest that the potential relevance of ADMA, SDMA, IL-6, and hsCRP to vascular aging may lie in their complementarity rather than in any single marker considered in isolation. However, current evidence does not establish that their combined assessment improves evaluation of vascular aging or cardiovascular risk beyond conventional approaches. Prospective studies are therefore needed to determine whether combined assessment of these biomarkers can improve identification of accelerated vascular aging beyond conventional risk assessment.
